# Acoustic waveguide with virtual soft boundary based on metamaterials

**DOI:** 10.1038/s41598-020-57986-9

**Published:** 2020-01-22

**Authors:** Guang-Sheng Liu, Yang Zhou, Ming-Hao Liu, Ying Yuan, Xin-Ye Zou, Jian-Chun Cheng

**Affiliations:** 1 Key Laboratory of Modern Acoustics, MOE, Institute of Acoustics, Department of Physics, Collaborative Innovation Center of Advanced Microstructures, Nanjing University, Nanjing, 210093 P. R. China; 2 State Key Laboratory of Acoustics, Chinese Academy of Sciences, Beijing, 100190 P. R. China; 3 School of Mathematics and Physics, Jiangsu University of Technology, Changzhou, 213001 P. R. China

**Keywords:** Materials science, Materials for devices, Structural materials, Acoustics

## Abstract

The use of acoustic metamaterials with novel phenomena to design acoustic waveguides with special properties has obvious potential application value. Here, we propose a virtual soft boundary (VSB) model with high reflectivity and half cycle phase loss, which consists of an acoustic propagation layer and an acoustic metamaterial layer with tube arrays. Then the waveguide designed by the VSB is presented, and the numerical and experimental results show that it can separate acoustic waves at different frequencies without affecting the continuity and the flow of the medium in the space. The VSB waveguide can enrich the functions of acoustic waveguides and provide more application prospects.

## Introduction

Acoustic metamaterials and metasurfaces are essentially the artificial structures with extraordinary acoustic properties. In general, these structures are placed in the open spaces of the incident acoustic fields to obtain the reflected and transmitted acoustic fields as required for different functions, such as broadband absorption with subwavelength structures^1–5^, high sound transmission^6–8^, phase modulation for focusing, deflection, cloaking vortexing and self-bending beams^9–14^, acoustic holographic imaging^15–17^. On the other hand, many works are aimed to manipulate acoustic waves through acoustic waveguides whose boundaries are constructed by unconventional acoustic structures. These approaches replace the conventional acoustic hard boundaries by the impedance boundaries with acoustic structures on the waveguide boundary. Such acoustic boundaries can be composed of phononic crystals to achieve waveguide function in the defect area^18–21^. By arranging different acoustic impedance structures on the waveguide boundary, functions such as acoustic insulation, acoustic absorption, and acoustic wave asymmetric transmission can be realized^22–24^. When applying piezoelectric materials with acoustic and electrical coupling property as the waveguide boundary, the external passive circuit can be used to freely control the dispersion of acoustic waves in the medium^25^. By arranging gradient changing acoustic metamaterials on the boundaries of the medium, acoustic rainbow trapping can also be achieved^26^. By arranging acoustic metasurfaces with acoustic wave deflection characteristics on both sides of the waveguide, it is possible to reflect acoustic waves twice at the upper and lower boundaries to achieve acoustic insulation in the pipe^27^.

**Figure 1:**
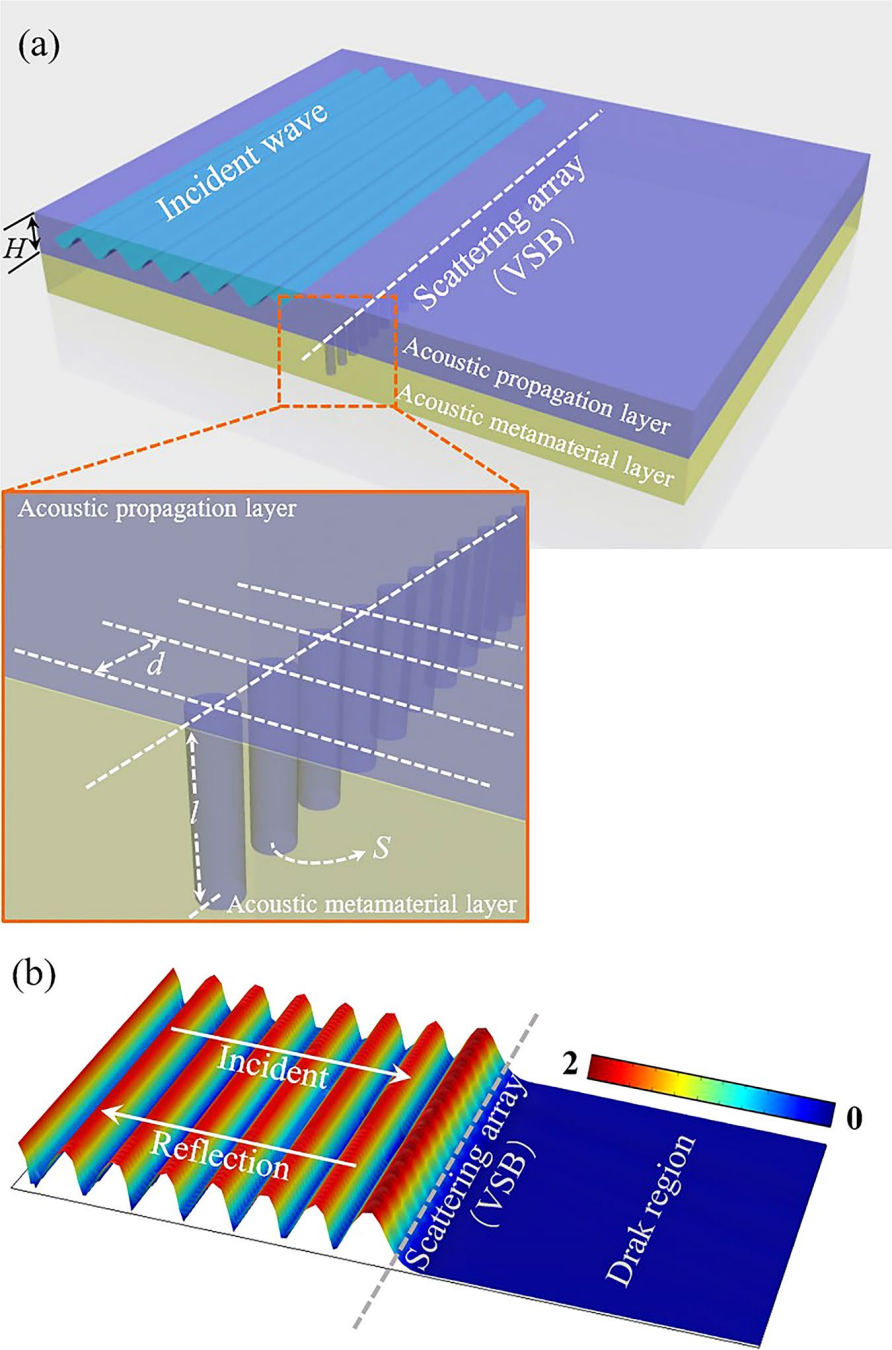
Schematic diagram of the VSB model. (**a**) The space is divided into the APL in blue and the AML in yellow. The acoustic waves in APL can be manipulated by the tube array in the AML. (**b**) The amplitude distribution of the acoustic field in the APL. A standing wave field is established on the reflection side, and a dark region is formed on the transmission side.

As mentioned above, the novel features of acoustic metamaterials have been used to construct waveguides with special functions. Nevertheless, these methods always encounter two problems: the acoustic structures placed in the space will hinder the exchange of medium between the waveguide and the external space; these structural units often become an acoustic hard boundary and lose the ability to control acoustic fields for the non-operating frequencies. Here, inspired by the previous methods and problems, we put forward a type of virtual soft boundary (VSB) based on an acoustic metamaterial layer consisted of the resonance tube unit array. Similar to the hard boundary, waves will be reflected at the soft boundary. But the difference is that the reflected waves will have a half cycle phase loss at the soft boundary. This phenomenon usually occurs when a wave enters a high refractive index medium from a low refractive index medium. This will cause the acoustic field distribution at the soft boundary to be different from the hard boundary. The most significant feature is that the acoustic pressure at the soft boundary is zero. The VSB model separates the space into two layers: the acoustic propagation layer (APL) in blue and acoustic metamaterial layer (AML) in yellow as shown in Fig. 1(a). Then a type of acoustic waveguide with the VSBs consisted of the different resonance tube unit arrays is presented. Since there is no physical boundaries in the APL of the VSB waveguide, it can separate acoustic waves at different frequencies without affecting the flow of the medium.

## Results

The schematic diagram of the VSB model is shown in Fig. 1. The bottom boundary of APL is directly connected with the AML, and the AML consists of the tube array as a scattering array. One end of each tube is open to the APL and the other end is closed. As we known, when the acoustic wavelength is 4 times the length of the short tube, the first-order resonance will occur in the short tube. Based on the lumped element model, the impedance of this short tube can be written as^28,29^:

1 $${Z}_{b}={R}_{b}-j\frac{{\rho }_{0}{c}_{0}}{S}\,\cot ({k}_{0}l),$$

where *R*_*b*_ is the acoustic resistance of the tube, (*ρ*_0_*c*_0_ / *S*)cot(*k*_0_*l*) is the acoustic reactance of the tube, *ρ*_0_, *c*_0_, *S*, *k*_0_ and *l* are the air density, acoustic velocity, the cross-sectional area of the tube, the wave number and the tube length, respectively. The acoustic damping of the short tube *R*_*b*_ is usually related to their cross-sectional area and length, and it can be calculated at different frequencies by different theories^30,31^. So it can be found that the damping can be controlled by adjusting the cross-sectional area of the small tube when the length of the tube is determined, and the impedance that determines the resonant frequency can be controlled by adjusting the length. As shown in Fig. 1(a), the thickness of APL is *H*, the medium in APL is fully continuous. AML is a functional layer designed by a scattering array consisted of short tube units, the distance between two adjacent tubes is *d*. The tube units in the AML will not affect the integrity and continuity of the medium in the APL, but can efficiently control the propagation of acoustic waves in the APL. For an infinitely long straight array, it can be divided into exactly the same parts, as shown in the magnified view in Fig. 1(a). The periodic unit can be treated as a simple lumped element in our model, and the corresponding acoustic field can be obtained. The incident acoustic pressure, the scattered acoustic pressure and the total acoustic pressure are defined as *P*_*i*_, *P*_*s*_ and *P*_*t*_, respectively. *P*_*i,a*_ and *P*_*s,a*_ are the corresponding acoustic pressure amplitudes. When the resonance occurs, the scattered acoustic pressure is:

2 $${P}_{{s}}=-\frac{{\rho }_{0}{c}_{0}}{2{R}_{b}dH+{\rho }_{0}{c}_{0}}{P}_{i,a}{e}^{j(\pm {k}_{0}x+\omega t)}\cdot $$

It can be found that the phase of scattered acoustic pressure is always opposite to the incident acoustic pressure, and the scattered acoustic pressure amplitude is related to the tube damping, the distance between the tubes, and the thickness of the APL. On the reflection side, since the direction of the scattered wave and the direction of incident wave are opposite, the *k*_0_ of the scattered wave has a positive sign. The total acoustic pressure is *P*_*t*_ = *P*_*i*_ + *P*_*s*_, so a standing wave field is established on the reflection side. On the transmission side, a negative sign is taken before *k*_0_, then the scattered wave and the incident wave have the same propagation direction, and *P*_*t*_ can be obtained as:

3 $${P}_{t}={P}_{i,a}(1-\frac{{\rho }_{0}{c}_{0}}{2{R}_{b}dH+{\rho }_{0}{c}_{0}}){e}^{j(-{k}_{0}x+\omega t)}.$$

It can be found that the acoustic waves on the transmission side is a superposition of two columns of acoustic waves that have opposite phases and propagate in the same direction. Then, by adjusting the parameters to obtain the condition $2{R}_{b}dH\ll {\rho }_{0}{c}_{0}$, *P*_*t*_ = 0 can be achieved. At this time, an acoustic boundary that can reflect acoustic waves efficiently with a half cycle phase loss without affecting the continuity and integrity of the medium has been formed. So far, the tube array in the AML can be regarded as the VSB for the APL.

In the numerical simulations with COMSOL Multiphysics and experiments, we take the specific parameters as: the tube length *l* = 18 *mm*, the tube section radius *a* = 2 *mm*, the dynamic viscosity coefficient *v* = 15.6 × 10^−6^*m*^2^/*s*, the air density *ρ*_0_ = 1.21*kg*/*m*^3^, the acoustic speed *c*_0_ = 343*m*/*s*^2^, the distance *d* = 6 *mm*, and the thickness *H* = 10 *mm*. So *R*_*b*_*dH* ≈ *ρ*_0_*c*_0_/10 can be obtained in the model. Then the amplitude distribution of the acoustic field in the APL with the normal incident situation is shown in Fig. 1(b). It can be found that the incident wave and the scattered wave are superimposed to form a standing wave field on the reflection side of the VSB, and to form a dark region on the transmission side of the VSB. For the other non-resonant frequencies, the wave will not be completely reflected at the VSB. At this situation, the VSB should be regard as an impedance boundary but not a soft boundary. So, when the non-resonant frequency waves pass through the impedance boundary, one part will be reflected and another part of the wave will transmit. The reflect wave and transmit wave will have a specific amplitude and phase based on the parameter of the impedance boundary. Nevertheless, it is clear that the resonant frequency wave will be totally reflected at the VSB, and this is the focus of our work.

**Figure 2:**
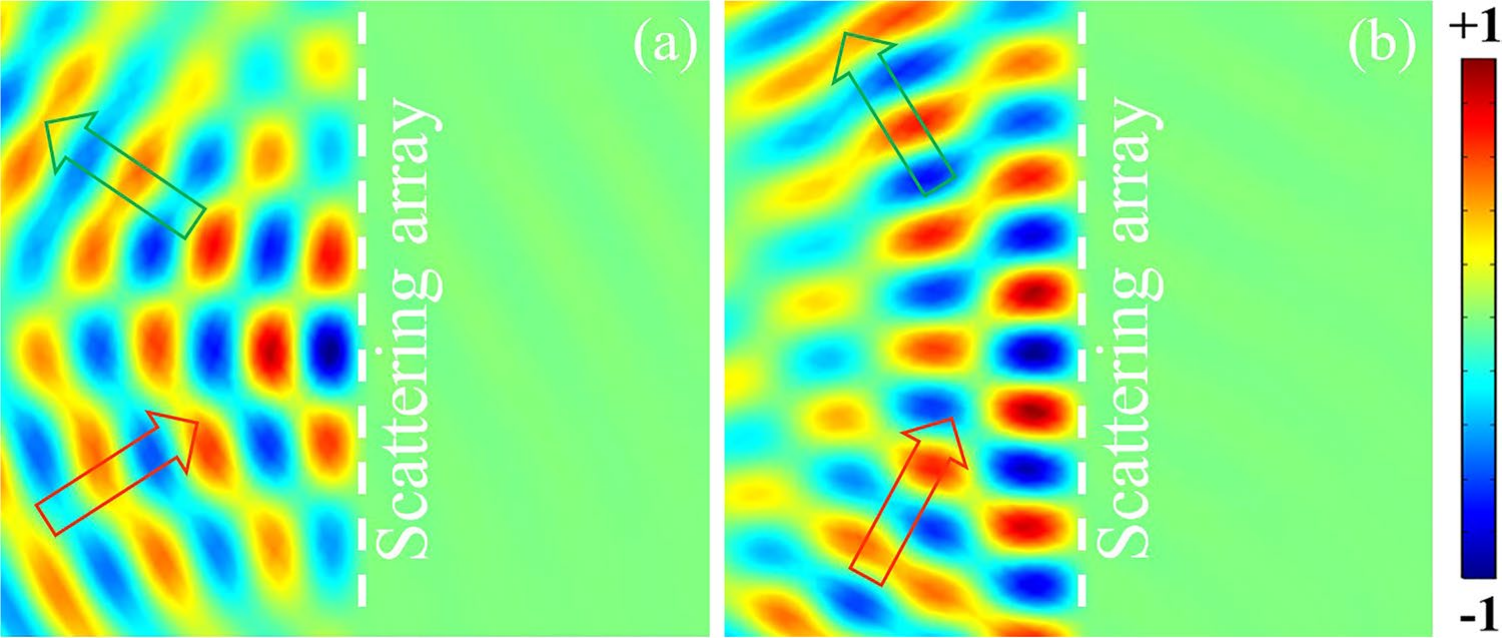
(**a**) Amplitude distribution of acoustic field for the 30° incident situation. (**b**) Amplitude distribution of acoustic field for the 60° incident situation.

In order to further verify the validity of the VSB, we also study the corresponding models of the 30° and 60°incident situation. Figure 2 shows the acoustic field when the incident angle is 30° and 60°. Similar to the normal incidence, the significant interference fringes can be seen on the reflection side, while the transmission side is still a dark region. This shows that the VSB still works well for the oblique incidence situation as long as the condition of $2{R}_{b}dH\ll {\rho }_{0}{c}_{0}$ is satisfied.

**Figure 3:**
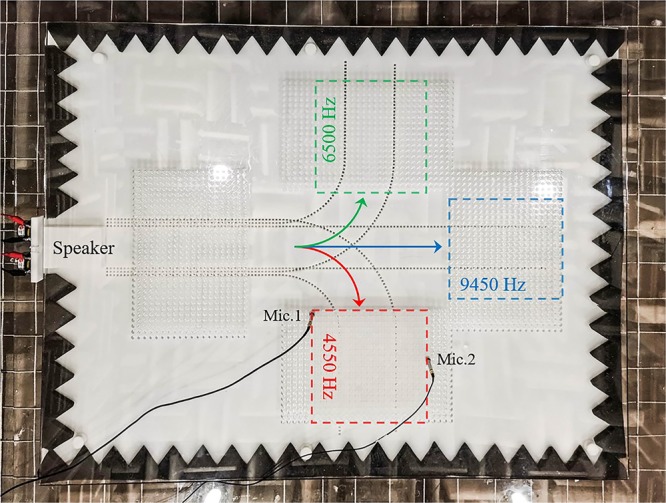
Schematic diagram and the experiment setting of the VSB waveguide. Acoustic waves are emitted into the waveguide by the left two speakers, and are separated into three channels corresponding to 4550 Hz, 6500 Hz and 9450 Hz, respectively. The red, green and blue squares are the measurement areas for the three frequencies, respectively. Two microphones are used to measure the acoustic pressure distribution in the corresponding areas.

So far, an efficient VSB model to control acoustic waves is established. Then we construct a type waveguide with the VSBs that can separate acoustic waves at different frequencies. As shown in Fig. 3, the VSB waveguide consists of three acoustic channels corresponding to three different frequencies. Each channel is built by two VSBs with tube arrays in the AML. The tubes belonging to different channels have different lengths corresponding to the resonant frequencies. As described above, the VSB only works at the resonant frequency of the scattering tube array in the AML, and does not affect the propagation of acoustic waves in the APL for non-resonant frequencies. It means that the three channels controlling acoustic waves of different frequencies can overlap with each other spatially without affecting with each other. In the VSB waveguide, the three channels coincide at the beginning and gradually separate into independent channels as the acoustic waves propagate. Here, we define the *x* as the direction of propagation and the *y* as the direction in which the standing wave is established for each channel. Thereupon, in the two-dimensional air layer, the acoustic pressure in the APL of each channel can be obtained as:

4 $$P=[A\,\cos ({{k}}_{{y}}{y})+{B}\,\sin ({{k}}_{{y}}{y})]{e}^{j(\omega t-{k}_{x}x)},$$

where *A* and *B* are the undetermined coefficients, *k*_*x*_ and *k*_*y*_ are wavenumbers in the two directions. As the previous analysis, the scattered wave on the VSB and the incident wave have the opposite phases. So, for a channel with width *L*, the boundary conditions at the upper and lower boundaries are ${P|}_{y=0}={P|}_{y=L}=0$. Then the acoustic pressure in one channel will be:

5 $$P={B}\,\sin (\frac{n\pi }{L}{y}){e}^{j(\omega t-{k}_{x}x)},\,n=0,1,\cdots \cdots \cdot $$

Moreover, the width of the waveguide needs to be satisfied with $L > (n\lambda /2)$ to obtain ${k}_{x} > 0$.

The experiment schematic of the waveguide to separate acoustic waves at three different frequencies is given in Fig. 3. The experiment was carried out in a thin air layer with the thickness of 10 mm. The upper and lower boundaries of the APL are composed of plexiglass and resin materials, respectively. The upper plexiglass plate has hundreds of holes for the microphone to measure the acoustic field, and the lower resin material plate (AML) is realized by 3D printing. In order to prevent reflections from the surrounding acoustic leakage, the cusp-shaped acoustic absorbing cotton is used to achieve the free field. The three channels of the VSB waveguide are composed of the scattering tube arrays with the tube lengths as ${l}_{1}=8mm$, ${l}_{2}=12mm$ and ${l}_{3}=18mm$, respectively. The cross section of the tube is a square with a side length of 4 *mm*. In the experiment, the resonant frequencies for the three different tube arrays are 9450 Hz, 6500 Hz and 4550 Hz, respectively. In order to satisfy the propagation conditions, the widths of the three channels for the three frequencies are: ${L}_{1}=80mm$, ${L}_{2}=92mm$ and ${L}_{3}=104mm$, respectively.

**Figure 4:**
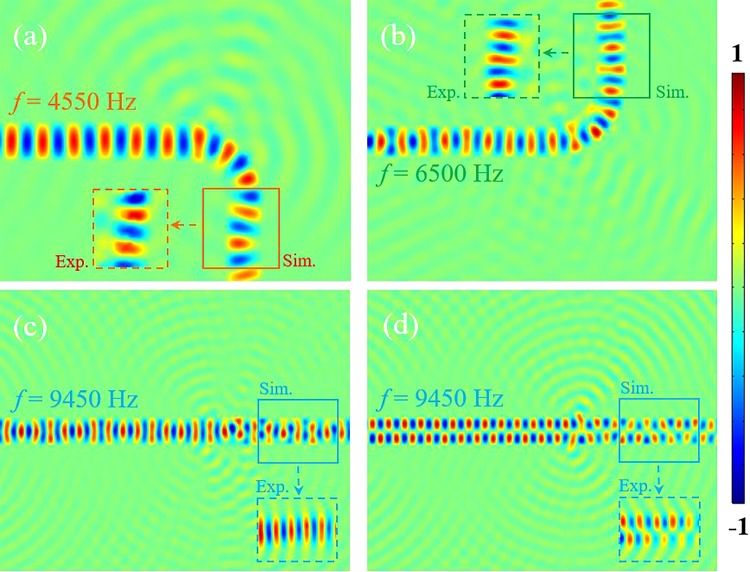
The numerical simulation and experimental results of the VSB waveguide. (**a**) The speaker emits 4550 Hz acoustic waves, the symmetrical wave propagates to the right along a given channel. (**b**,**c**) give the results for 6500 Hz and 9550 Hz, respectively. (**d**) Antisymmetric propagating wave is created by exciting the two speaker with opposite phase signal, and it propagates alone the established channel.

**Figure 5:**
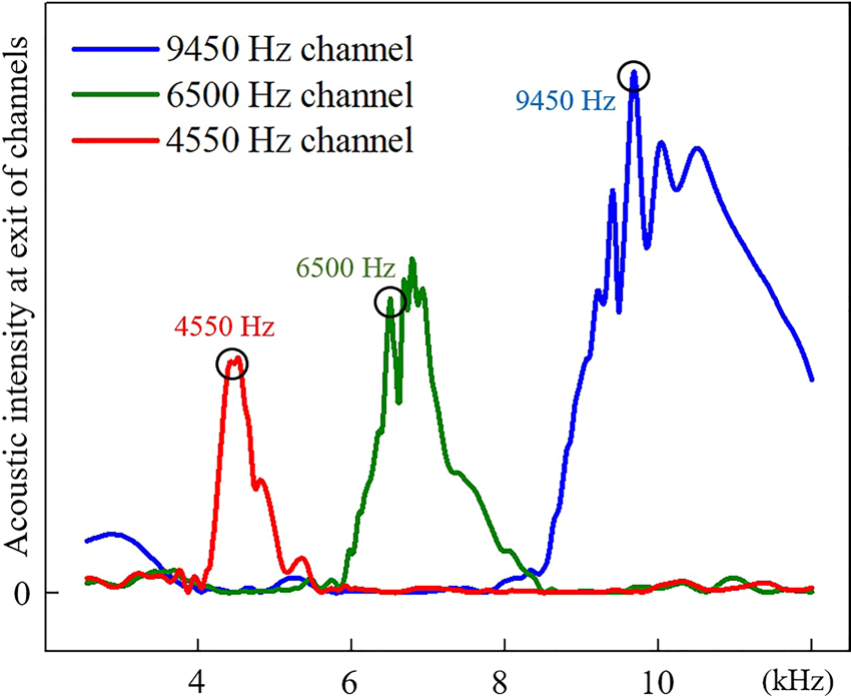
The numerical results of acoustic intensity at the exits of three channels with non-monochromatic incident.

Figure 4 shows the numerical simulation and experimental results in each channel of the VSB waveguide at different frequencies. The parameter settings in the simulation are the same as in the experiment. In the finite element simulation, we consider the thermal viscous effect of air in the numerical simulation. At the same time, three measuring regions of 4550 Hz, 6500 Hz and 9450 Hz in the Fig. 3 are marked in red, green and blue box regions. Figure 4(a–c) show the propagation of the first order symmetrical waves of the three frequencies in the VSB waveguide, respectively. The simulation and experimental results verified that the every channel of the waveguide based on the VSBs can efficiently control the acoustic waves at the pre-set frequency without affecting other frequencies. In Fig. 5, We calculated the acoustic intensity at the exits of the three channels after the non-monochromatic signals entered the waveguide. The three color curves represent the acoustic intensity at the different exits. It is obviously to find the separation of different frequencies. In order to further verify the robustness of the waveguide, the first-order antisymmetric mode of 9450 Hz is also studied and the corresponding results are shown in Fig. 4(d). According to the Eq. (5), the first-order antisymmetric mode can be achieved by the two opposite phase signals which can be excited by the two speakers. It is worth pointing out that the antisymmetric wavefront which have a dipole form has a stronger tendency to leak than the symmetric mode in polar axis direction, but the result shows that it can also spread over a long distance in the VSB waveguide. So the above results show that the VSB waveguide has a wide applicability and can control different modes of acoustic waves efficiently.

## Discussion

In summary, the VSB model based on the acoustic metamaterials with tube arrays is established to control acoustic waves. The VSB can reflect acoustic waves efficiently with a half cycle loss. Compared with the traditional acoustic boundaries, the structure constituting the VSB is outside the wave propagation layer, thus the medium continuity in the propagation layer is not interfered by the VSB. Since the VSB consists of a periodically arranged resonant structures, it is only “visible” at the designed resonant frequency, and is “invisible” for other frequencies. We numerically and experimentally study the waveguide with VSB design, which can separate acoustic waves at different frequencies without affecting the flowing of the medium in the propagation layer. Based on the efficient and stable performance in simulation and experiment results for the symmetrical mode and the antisymmetric mode, we believe that VSB can provide a solution for constructing acoustic soft boundaries and implementing complex functional acoustic waveguides, and the VSB waveguide can enrich the functions of acoustic waveguides and provide more application prospects, such as constructing an visual acoustic wall in microfluidic systems.

## Methods

### Numerical simulations

Throughout the paper, the numerical simulations are conducted by the finite element method based on commercial software COMSOL Multiphysics. The background medium is air whose mass density, dynamic viscosity and sound speed are ${\rho }_{0}=1.21kg/{m}^{3}$, $v=15.6\times {10}^{-6}{m}^{2}/s$ and ${c}_{0}=343m/s$, respectively. The viscous effect in air at narrow area provided by the thermoacoustics module in the COMSOL Multphysics.

### Acoustic measurements

The measurement is performed in the anechoic chamber in order to eliminate the undesired reflected waves. Two 1/4-inch microphone is used for measuring the acoustic field in three measurement field which marked red, green and blue. Two individually controlled loud speakers for exciting symmetrical and antisymmetric modes acoustic waves. Acoustic absorbing foams are also set around the experimental area. Mechanical parameters of AML which consist of ABS plastic are mass density ${\rho }_{A}=1180kg/{m}^{3}$ and sound speed ${c}_{A}=2700m/s$.

## References

[1] Zhang C, Hu X (2016). Three-dimensional single- port labyrinthine acoustic metamaterial: perfaect absorption with large bandwidth and tunability. Phys. Rev. Appl..

[2] Yang M, Chen S, Fu C, Sheng P (2017). Optimal sound-absorbing structures. Mater. Horiz..

[3] Li J, Wang W, Xie Y, Popa B, Cummer SA (2016). A sound absorbing metasurface with coupled resonators. Appl. Phys. Lett..

[4] Ma G, Yang M, Xiao S, Yang Z, Sheng P (2014). Acoustic metasurface with hybrid resonances. Nat. Mater..

[5] Mei J (2012). Dark acoustic metamaterials as super absorbers for low-frequency sound. Nat. Commun..

[6] Cheng Y (2015). Ultra-spares metasurface for high reflection of low-frequency sound based on artificial Mie resonances. Nat. Mater..

[7] Shen C, Xu J, Fang NX, Jing Y (2014). Anisotropic complementray acoustic metamaterial for canceling out aberrating layers. Phys. Rev. X.

[8] Liang Z, Li J (2012). Extreme acoustic metamaterial by coiling up space. Phys. Rev. Lett..

[9] Li Y (2014). Experimental realization of full control of reflected waves with subwavelength acoustic metasurfaces. Phys. Rev. Applied.

[10] Lan J, Li Y, Xu Y, Liu X (2017). Manipulation of acoustic wavefront by gradient metasurface based on Helmholtz Resonators. Sci. Rep..

[11] Li Y, Liang B, Gu Z, Zou X, Chen J (2013). Reflected wavefront manipulation based on ultrathin planar acoustic metasurfaces. Sci. Rep..

[12] Dubois M, Shi C, Wang Y, Zhang X (2017). A thin and conformal metasurface for illusion acoustics of rapidly changing profiles. Appl. Phys. Lett..

[13] Jiang X, Li Y, Liang B, Chen J, Zhang L (2016). Convert acoustic resonances to orbital angular momentum. Phys. Rev. Lett..

[14] Zhang P (2014). Generation of acoustic self-bending and bottle beams by phase engineering. Nat. Commun..

[15] Melde K, Mark AG, Qiu T, Fischer P (2016). Holograms for acoustics. Nature..

[16] Zhu Y (2018). Fine manipulation of sound via lossy metamaterials with independent and arbitrary reflection amplitude and phase. Nat. Commun..

[17] Xie Y (2016). Acoustic holographic rendering with two-dimensional metamaterial-based passive phased array. Sci. Rep..

[18] Ma PS, Kwon YE, Kim YY (2013). Wave dispersion tailoring in an elastic waveguide by phononic crystals. Appl. Phys. Lett..

[19] Assouar MB, Senesi M, Oudich M, Ruzzene M, Hou Z (2012). Broadband plate-type acoustic metamaterial for low-frequency sound attenuation. Appl. Phys. Lett..

[20] Khelif A, Rouhani BD, Vasscur JO, Deymier PA (2003). Transmission and dispersion relations of perfect and defect-containing waveguide structures in phononic band gap materials. Phys. Rev. B..

[21] Otsuka PH (2013). Broadband evolution of phononic-crystal-waveguide eigenstates in real- and k-spaces. Sci. Rep..

[22] Merkel A, Theocharis G, Richoux O, Garcia VR, Pagneux V (2015). Control of acoustic absorption in one-dimensional scattering by resonant scatterers. Appl. Phys. Lett..

[23] Long H, Cheng Y, Liu X (2017). Asymmetric absorber with multiband and broadband for low-frequency sound. Appl. Phys. Lett..

[24] Fu C, Zhang X, Yang M, Xiao S, Yang Z (2017). Hybrid membrane resonators for multiple frequency asymmetric absorption and reflection in large waveguide. Appl. Phys. Lett..

[25] Casadei F, Delpero T, Bergamini A, Ermanni P, Ruzzene M (2012). Piezoelectric resonator arrays for tunable acoustic waveguides and metamaterials. J. Appl. Phys..

[26] Zhu J (2013). Acoustic rainbow trapping. Sci. Rep..

[27] Zhang HL, Zhu YF, Liang B, Yang J, Cheng JC (2017). Sound insulation in a hollow pipe with subwavelength thickness. Sci. Rep..

[28] Cheng, J. C. *Acoustic Principle* (Science Press, Beijing, 2012), pp. 387–389

[29] Zhu Y (2015). Dispersionless Manipulation of reflected acoustic wavefront by subwavelength corrugated surface. Sci. Rep..

[30] Cheng, J. C., *Acoustic Principle* (Science Press, Beijing, 2012), pp. 541–547

[31] Maa D (1998). Potential of microperforated panel absorber. J. Acoust. Soc. Am..

